# A Multichannel Fluorescent Array Sensor for Discrimination of Different Types of Drug-Induced Kidney Injury

**DOI:** 10.3390/s23136114

**Published:** 2023-07-03

**Authors:** Kunhui Sun, Bing Wang, Jiaoli Lin, Lei Han, Meifang Li, Ping Wang, Xiean Yu, Jiangwei Tian

**Affiliations:** 1 State Key Laboratory of Natural Medicines, Jiangsu Key Laboratory of TCM Evaluation and Translational Research, School of Traditional Chinese Pharmacy, China Pharmaceutical University, Nanjing 211198, China; sunkunhuilst@163.com (K.S.); lin97007@126.com (J.L.); hanhllei@163.com (L.H.); 2 NMPA Key Laboratory for Bioequivalence Research of Generic Drug Evaluation, Shenzhen Institute for Drug Control, Shenzhen 518057, China; wangbingszyj@163.com (B.W.); szlimeifang@126.com (M.L.); wangping662@sina.com (P.W.)

**Keywords:** multichannel fluorescent array sensor, drug-induced kidney injury, Au nanoparticles–polyethyleneimine (AuNPs–PEI), fluorophore-labeled proteins

## Abstract

The differences in urinary proteins could provide a novel opportunity to distinguish the different types of drug-induced kidney injury (DIKI). In this research, Au nanoparticles–polyethyleneimine (AuNPs–PEI) and the three fluorophore-labeled proteins (FLPs) have been constructed as a multichannel fluorescent array sensor via electrostatic interaction, which was used to detect the subtle changes in urine collected from the pathological state of DIKI. Once the urine from different types of DIKI was introduced, the binding equilibrium between AuNPs–PEI and FLPs would be broken due to the competitive binding of urinary protein, and the corresponding fluorescence response pattern would be generated. Depending on the different fluorescence response patterns, the different types of DIKI were successfully identified by principal component analysis (PCA) and linear discriminant analysis (LDA). Accordingly, the strategy was expected to be a powerful technique for evaluating the potential unclear mechanisms of nephrotoxic drugs, which would provide a promising method for screening potential renal-protective drugs.

## 1. Introduction

Kidney is an essential organ of the human body and plays a very important role in regulating fluid homeostasis, material excretion and maintaining electrolyte balance. Due to its relatively rich blood supply and concentrated excretion of drugs, it has become one of the most vulnerable organs [1,2]. Drug-induced kidney injury (DIKI) refers to adverse reactions of the kidney induced by therapeutic doses of drugs and toxic reactions owing to excessive or unreasonable use of drugs [3]. In addition, DIKI was a serious complication in hospitalized patients with a higher possibility of developing progressive chronic kidney disease or end-stage kidney disease [4]. Meanwhile, it was estimated that up to 25% of hospitalizations have been specifically attributed to DIKI owing to acute renal failure, and the incidence was even as high as 60% in older patients [3,5]. Therefore, DIKI is an important cause of morbidity and mortality in hospitalized patients, as well as a major obstacle for drug development.

The pathophysiological mechanisms of DIKI are complex, and the mechanism would vary with different drugs or drug categories [6]. For example, anti-prostaglandin drugs or anti-angiotensin II drugs could interfere with renal self-regulation of glomerular pressure and decrease glomerular filtration rate by changing the balance between vasoconstriction and expansion, thus affecting hemodynamic changes and leading to renal injury [7,8]; aminoglycoside antibiotics had a direct toxic effect on renal tissue especially in proximal tubules, causing a series of cascading damage reactions such as mitochondrial damage, lysosomal rupture or enhanced oxidative stress, which induced renal injury [9]; proton pump inhibitors could be deposited in the renal interstitium as antigens or haptens, inducing immune responses and cause inflammatory damage [10]. Therefore, understanding the mechanisms of DIKI and drug-related risk factors could predict and detect renal damage at an earlier stage, as well as reduce the risk of renal failure.

In previous clinical practice, there was the ongoing dependence on traditional biomarkers such as blood urea nitrogen (BUN) and/or serum creatinine (Scr) to diagnose DIKI [11]. Although theses biomarkers were considered as diagnostic indicators in routine care, they were also affected by several non-kidney-related factors such as nutritional status, muscle mass, age and sex, leading to inaccuracy and low hysteresis quality for diagnosis [12]. In comparison, the clusterin, neutrophil gelatinase-associated lipocallipin (NGAL), Cystatin C and N–acetyl–β–glucosaminidase (NAG) were selected as the novel biomarkers for urinalysis, which were more sensitive and reliable than traditional diagnostic methods [13,14]. However, there were also limited methods for the detection of these biomarkers, usually using commercial immunoassays and matrix-assisted laser desorption/ionization time-of-flight mass spectrometry (MALDI–TOF–MS). The disadvantages of commercial immunoassays for novel biomarkers were cumbersome operation, while the application of MALDI–TOF–MS for these biomarkers was also limited by the number of reference mass spectra available in the database [15]. In addition, the above-mentioned methods could only be used to judge the occurrence and development of DIKI, so the complex injury mechanisms of DIKI may be difficult to discriminate. Therefore, a novel, sensitive and specific detection method is urgently needed to realize the low cost and noninvasive detection of the different types of DIKI.

Exploring the physiological function of the kidney further, proteinuria was a typical symptom of renal disease, which was a comprehensive manifestation of impaired renal function caused by abnormal glomerular filtration function or renal tubular reabsorption [16]. The glomerular filtration barrier was composed of a glomerular basement membrane, endothelial cell and podocytes. Under normal physiological conditions, it can freely filter positively charged proteins with particle size less than 8 nm [17,18]. However, under pathological conditions, the glomerular filtration system was destroyed, leading to significant changes in the type and content of protein in filtered urine. Indeed, according to the investigation, proteinuria was also present in the clinical manifestations of different types of DIKI. For example, aminoglycoside antibiotics could destroy lysosomes by interfering with normal function of mitochondria and promote the formation of oxygen free radicals leading to increased oxidative stress, resulting in apoptosis and necrosis of renal tubular epithelial cells. Hence, the renal tubular reabsorption function was reduced, thus affecting the generation of proteinuria [19,20], while antibacterial drugs such as rifampicin could act as the hapten to combine with macromolecular substances in the blood to form an antigen–antibody complex, leading to glomerulonephritis or acute interstitial nephritis with clinical manifestations of hematuria, which could increase the mildly moderate albuminuria [21,22]. Therefore, based on the different injury mechanisms and time–effect relationship of drugs, differences in proteinuria may bring new opportunities for the identification of different types of kidney injury.

The “chemical nose/tongue” sensor array was a powerful molecular recognition strategy inspired by the human olfactory system to identify complex bioanalytes. Unlike the traditional antibody-based “lock key” recognition mode, in the “nose” approach, differential interactions between analytes and receptor array would produce a specific pattern for identification [23,24]. In recent years, this strategy has been used to detect various biological analytes such as proteins [25,26], bacteria [27] and cells [28,29]. For example, Xu et al. [30] designed a sensor array based on Au NCs functionalized with six kinds of near-infrared fluorescent double ligands. Based on this sensor, the authors were able to identify 10 different proteins and successfully distinguish serums from healthy people at different stages of breast cancer. In addition, Edward and co-authors [31] demonstrated a sensor array based on eight dual-ligand-functionalized gold fluorescent nanodots with diverse surface properties. The authors used the sensor array to successfully identify eight different proteins, providing 100% accuracy for the unknowns. Accordingly, in view of the development characteristics of proteinuria from different types of DIKI, the construction of a new rapid detection method based on multichannel fluorescent array sensor technology was helpful for the identification of different types of DIKI.

**Figure 1:**
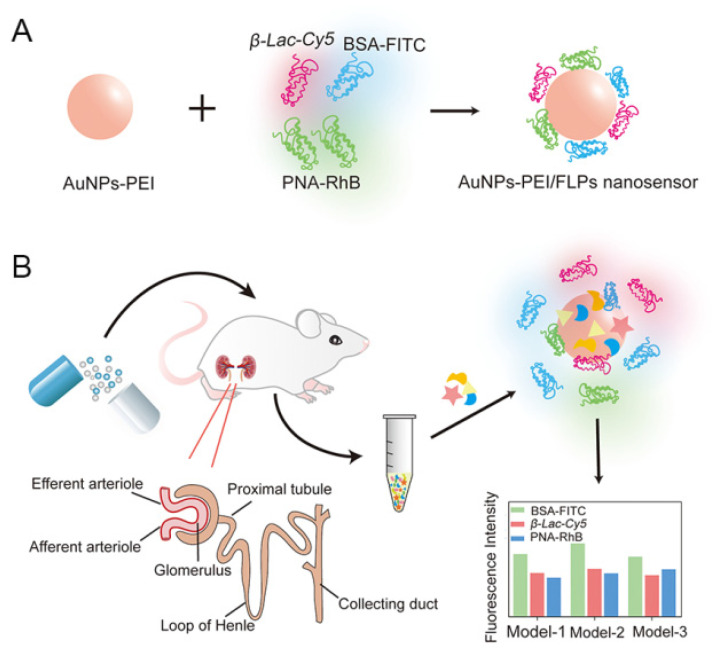
Schematic illustration of (**A**) structure of AuNPs–PEI/FLPs and (**B**) a multichannel sensor array that instantaneously identifies different types of DIKI.

In this study, we employed a multichannel fluorescent array sensor to identify different types of DIKI via detecting the differences in proteinuria induced by multiple nephrotoxic drugs. The sensor was composed of gold nanoparticles–polyethyleneimine (AuNPs–PEI) copolymer and three fluorescence-labeled proteins (FLPs) whose fluorescence was initially quenched by AuNPs–PEI due to fluorescence resonance energy transfer (FRET) but recovered upon the addition of proteinuria (Figure 1). The AuNPs–PEI copolymer with excellent quenching property and positive charge was prepared via the one-step method. FLPs were three proteins with various molecular weights labeled with fluorophores with different emission wavelengths, namely, bovine serum albumin–fluorescein isothiocyanate (BSA–FITC), peanut agglutinin–rhodamine B (PNA–RhB) and β–lactoglobulin–cyanine dye Cy5 (β–Lac–Cy5), which were adsorbed on the surface of AuNPs–PEI through electrostatic interaction. During the development of kidney injury induced by nephrotoxic drugs, the difference in protein in terms of types and content in urine caused by different degrees of kidney injury would induce the disassociation of fluorescent signal molecules from the sensor, generating a unique fluorescent signal response pattern, which would reflect the process of DIKI. Furthermore, the time–effect relationship of drug-induced renal injury was introduced and expanded the signal variables, further achieving the accurate identification of three types of DIKI. Therefore, this work provided a novel strategy for the accurate detection of the progression of kidney injury and the evaluation of the potential mechanisms of DIKI.

## 2. Materials and Methods

### 2.1. Chemicals and Reagents

Gold acid chloride trihydrate (HAuCl_4_·3H_2_O) was obtained from Aladdin Bio-Chem Technology Co., Ltd. (Shanghai, China). Polyethyleneimine (PEI, Mw = 1800) was purchased from Macklin Biochemical Co., Ltd. (Shanghai, China). Three fluorescent signal molecules (BSA–FITC, PNA–RhB and β–Lac–Cy5) were synthesized by Bersee Science and Technology Co., Ltd. (Beijing, China) and Fubo Biotechnology Co., Ltd. (Beijing, China). Ibuprofen and diclofenac sodium were supplied by Aladdin Co., Ltd. (Shanghai, China). Omeprazole, lansoprazole, esomeprazole, pantoprazole, gentamicin, tobramycin and neomycin were provided by Yuanye Biotechnology Co., Ltd. (Shanghai, China). Naproxen was supplied from Solarbio Life Sciences Co., Ltd. (Beijing, China). Ultrapure water was prepared by using the Millipore Simplicity System (Millipore, Bedford, MA, USA) with a resistivity of 18.2 MΩ·cm^−1^.

### 2.2. Instruments

The absorption spectra of AuNPs, PEI and AuNPs–PEI were performed on a Cary Series UV–vis spectrophotometer (Agilent Technologies, Santa Clara, CA, USA). Bruker Tensor 27 FTIR spectrometer (Bruker, Ettlingen, Germany)was used to perform Fourier-transform infrared spectra of AuNPs, PEI and AuNPs–PEI (resolution: better than 0.25 cm^−1^; signal-to-noise ratio: better than 50,000:1; spectral area: 8000~350 cm^−1^ (basic), 15,500~20 cm^−1^ (optional); accuracy: 0.005 cm^−1^). The excitation and emission spectra were acquired on a Cary Eclipse Fluorescence Spectrophotometer (Agilent Technologies Inc. USA) (resolution: 1.5 nm; the wavelength range: 200–900 nm) with a slit width of 5 nm. The morphology of AuNPs–PEI was featured using a JEOL JEM–200CX transmission electron microscope (TEM) operating at 200 KV (JEOL, Tokyo, Japan). Mastersizer 2000 particle size analyzer (Malvern Instruments Ltd., Worcestershire, UK) was used to measure the average size of AuNPs, AuNPs–PEI and AuNPs–PEI/FLPs sensor by dynamic light scattering (DLS). Zeta potential was tested on a Zeta sizer (Nano–Z, Malvern, UK). The fluorescence intensity was obtained on Varioskan Flash (Thermo Scientific, USA) HE staining images were obtained using NanoZoomer 2.0 RS (Hamamatsu, Hamamatsu city, Japan) in a digital pathology slice scanner.

### 2.3. Preparing of AuNPs–PEI/FLPs Sensor

According to previous study, AuNPs–PEI can be prepared from HAuCl_4_ by using PEI as reducing agent and stabilizer. In brief, 10 mg PEI was dissolved in 1 mL of ultrapure water. Then, 125 μL 1% HAuCl_4_·3H_2_O was absorbed and diluted into 2.5 mL ultrapure water, and then 100 μL PEI was slowly added; then, the mixture was stirred on a magnetic stirrer to react fully for 8 h at room temperature without light. After stirring, the color of the reaction solution changed from yellow to dark red, and AuNPs–PEI was preliminarily obtained. Finally, the solution was filtered with 0.22 μm cellulose ester membrane and stored in a refrigerator at 4 °C for further experiments. In order to prepare the AuNPs–PEI/FLPs sensor, the prepared AuNPs–PEI polymer was fully mixed with FLPs of equal molar ratio to form AuNPs–PEI/FLPs sensor through electrostatic interaction.

### 2.4. Fluorescence Titrations

In order to obtain the optimal binding ratio of AuNPs–PEI and FLPs, the fluorescence titration experiment was performed to determine the quenching efficiency. AuNPs–PEI with different volumes was added into a single FLP (0.015 nM each) to measure the corresponding fluorescence intensity. The excitation/emission wavelengths of BSA–FITC, PNA–RhB and β–Lac–Cy5 were 495/520, 550/575 and 654/667 nm, respectively. Then, the quenching efficiency was obtained as follows: Quenching efficiency (%) = 100 × (F0 − Fn)/F0, where F0 represented the fluorescence intensity of FLPs without AuNPs–PEI, and Fn was the fluorescence intensity after adding various volumes of AuNPs–PEI to FLPs. Finally, nonlinear least-squares curve fitting analysis was used to calculate the binding constant (Ka) via a 1:1 binding model.

The fitting of quenching data was performed by using the following Stern–Volmer equation

I0/I[Q] = 1 + Ksv[Q]

I0 and I[Q] represent the fluorescence intensity of the system in the absence and presence of AuNPs–PEI, respectively, and Ksv is the quenching constant, [Q] is the concentration of AuNPs–PEI.

### 2.5. Animals and Experimental Design

Healthy male ICR mice weighing 20–24 g were purchased from the Experimental Animal Center and Institute of Comparative Medicine of Yangzhou University. The animals were raised in a standard environment at a temperature of 23 ± 2 °C, light/dark cycle for 12 h, and had free access to food and water. They were adapted to environmental conditions for at least 7 days prior to formal experiments. The experimental design contained one control group and three drug model groups, as follows:

(1) Normal control group. Mice received saline.
(2) Aminoglycoside antibiotics (AGs) group. Gentamicin (97.07 mg/kg/d), tobramycin (46.41 mg/kg/d), neomycin (303 mg/kg/d).
(3) Proton pump inhibitor (PPI) group. Omeprazole (6.07 mg/kg/d), lansoprazole (4.55 mg/kg/d), esomeprazole (6.07 mg/kg/d), pantoprazole (6.07 mg/kg/d).
(4) Non-steroidal anti-inflammatory (NSAIDs) group. Ibuprofen (91.0 mg/kg/d), diclofenac sodium (15.2 mg/kg/d), naproxen (121.3 mg/kg/d).

Drugs were given by gavage at a fixed time every day for 14 days. The administered dose is the maximum clinical dose converted to mouse dose. Urine of mice in each group was collected by bladder extrusion method on 0, 1, 3, 5, 7, 9, and 14 days. Kidney tissues of mice were collected for subsequent use.

### 2.6. Fluorescence Responses Assay of Detection and Identification of Proteinuria

The collected urine samples were centrifuged at 3000 rpm for 10 min. Firstly, 190 μL of AuNPs–PEI/FLPs sensor was added at the optimal ratio on a 96-well plate, and then 10 μL of mice urine supernatants of different administration days was drawn into it, and the solution was mixed. Fluorescence intensity was read by fluorescence microplate reader, and the SIMCA–P 14.1 and SPSS 22.0 software were used for principal component analysis (PCA) and linear discriminant analysis (LDA) for statistical analysis.

### 2.7. Histopathological Analysis

The kidney tissues were stored in 10% formaldehyde immediately after killing. All the samples were embedded in paraffin and slices with a thickness of 5 µm were prepared. The slices were stained with hematoxylin–eosin (HE) for histological examination.

### 2.8. Statistical Analysis

Data were presented as mean ± SD. All experimental parameters were tested at least three times. Statistical analyses were performed using GraphPad Prism, Origin, SIMCA–P 14.1, SPSS 22.0 software. *p* < 0.05 was construed as statistical significance.

## 3. Results

### 3.1. Characterization of AuNPs–PEI/FLPs Sensor

**Figure 2:**
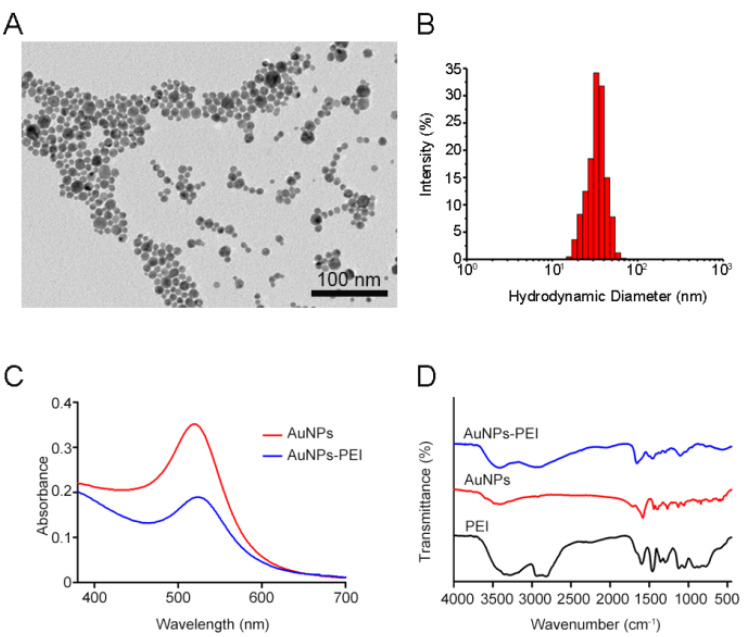
(**A**) TEM image of AuNPs–PEI. (**B**) Hydrodynamic size of AuNPs–PEI determined by DLS. (**C**) Absorption spectrum of AuNPs and AuNPs–PEI. (**D**) FTIR spectroscopy of PEI, AuNPs and AuNPs–PEI.

The AuNPs–PEI was prepared from HAuCl_4_ by using PEI as reducing agent and stabilizer, and the traditional citric acid reduction method was also employed to synthesize AuNPs [32]. The TEM results showed that the prepared AuNPs–PEI was spherical and uniformly dispersed (Figure 2A). The TEM of AuNPs was displayed in Figure S1. The DLS data of AuNPs and AuNPs–PEI showed that the average hydrodynamic diameter increased from 24.65 nm (Figure S2) to 35.45 nm (Figure 2B), which proved the successful preparation of AuNPs–PEI. Furthermore, the average size of AuNPs–PEI/FLPs sensor was 43.20 nm (Figure S3), indicating that the FLPs were bound to the surface of AuNPs–PEI. Figure 2C shows the UV–Vis absorption spectra of AuNPs and AuNPs–PEI, respectively. The maximum absorption peak of AuNPs was at 521 nm, while that of AuNPs–PEI was at 525 nm, showing a red shift, which further verified the successful synthesis of AuNPs–PEI. Meanwhile, FTIR provided additional insight into the process of synthesizing of AuNPs–PEI. The most prominent FTIR spectra of AuNPs–PEI were characterized by the bands at 1631 cm^−1^ and 1557 cm^−1^, which were preliminarily identified as C=O stretching and N–H bending of the amide group, respectively (Figure 2D). In addition, the Zeta potential results showed that the negative charge of AuNPs was −32.6 mV (Figure S4), while the positive charge of AuNPs–PEI was +21.9 mV (Figure S5) with the sensor potential of −9.49 mV (Figure S6), indicating that AuNPs–PEI and FLPs were bonded by electrostatic interaction. And, the sensor had good stability, which was reflected in the changes in hydrodynamic diameter and Zeta potential within 2 weeks (Figures S7 and S8). In summary, all the above data indicated that AuNPs and AuNPs–PEI/FLPs sensor were successfully prepared.

### 3.2. Fluorescence Titrations

**Figure 3:**
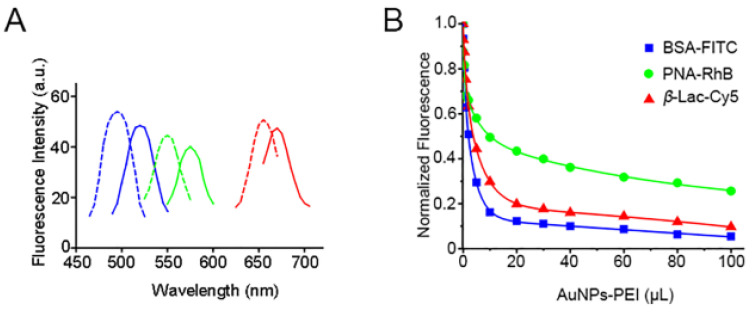
(**A**) Excitation and emission wavelengths of three FLPs. The blue, green and red lines represent the fluorescence spectra of BSA–FITC, PNA–RhB and β–Lac–Cy5, respectively. The dotted line represents the excitation spectrum and the solid line represents the emission spectrum. (**B**) Fluorescence titration of the three FLPs.

The excitation and emission fluorescence spectra of the FLPs (BSA–FITC, PNA–RhB, β–Lac–Cy5) showed that none of their spectra overlapped or influenced each other, which could be used in the application of a multichannel fluorescent array sensor (Figure 3A and Figure S9–S11). In addition, the fluorescence quenching process of the FLPs was very rapid, and the equilibrium state could be reached in only 30 s (Figure S12). The fluorescence titration experiments were performed to obtain the binding parameters of AuNPs–PEI towards three FLPs. Using nonlinear least square fitting curve to analyze the corresponding binding constants between each FLP and AuNPs–PEI (Figure 3B), there was the differential and high affinity required for multi-channel output. The results showed that the binding affinity of AuNPs–PEI to different FLPs was FLP dependent in the order of BSA–FITC (1.41 × 10^8^ M^−1^) > β–Lac–Cy5 (7.43 × 10^7^ M^−1^) > PNA–RhB (2.68 × 10^6^ M^−1^) (Table S1) with an order of magnitude difference, indicating that the competitive combination between the sensor and various analytes in proteinuria. Moreover, the fluorescence quenching reached saturation when the volume of AuNPs–PEI was 80 μL. Therefore, 80 μL AuNPs–PEI and 0.015 nM FLP were selected in the subsequent experiments.

Moreover, the fluorescence intensity of the AuNPs–PEI/FLPs sensor was almost unchanged in the pH range of 4.5–8.5 (pH of normal human urine) (Figures S13–S15). These results showed that the AuNPs–PEI/FLPs sensor had stable fluorescence performance in the urine environment. Therefore, the AuNPs–PEI/FLPs sensor was relatively stable in the urine environment and would not be affected by the pH of the urine, and the effect of inorganic ions in urine on the fluorescence signal of AuNPs–PEI/FLPS sensor was also investigated. As shown in Figures S16–S18, after adding ionic mixture (including Na^+^, K^+^, Mg^2+^ et al.), the fluorescence intensity of AuNPs–PEI/FLPs sensor was stable. In conclusion, these results suggested that the sensing system was suitable for the application in urine.

### 3.3. Discrimination and Evaluation of the Process of AGs Nephropathy Model

**Figure 4:**
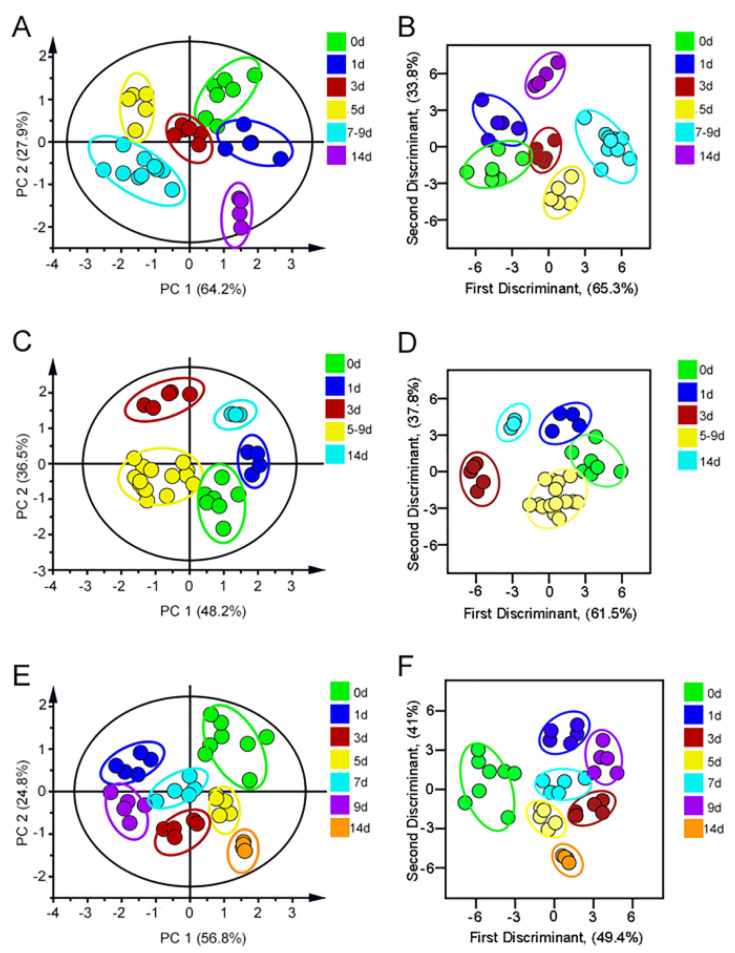
(**A**) The PCA score plot and (**B**) LDA score of Gentamicin-induced kidney injury. (**C**) The PCA score of Neomycin-induced kidney injury. (**D**) The LDA score of Neomycin-induced kidney injury. (**E**) The PCA score plot and (**F**) LDA score of Tobramycin-induced kidney injury.

Gentamicin, neomycin and tobramycin were selected as AGs for testing. The urine of mice after administration of different days was mixed with AuNPs–PEI/FLPs (optimal ratio) sensor to collect fluorescence signals at the optimal excitation wavelength, which served as a unique fluorescence fingerprint of different drug injury process. The fluorescence intensity of three FLPs in AGs-induced kidney injury is shown in Table S2. In addition, principal component analysis (PCA) was used to perform dimensionality reduction to convert multiple variables into fewer comprehensive variables to identify the fluorescence response patterns of different damage processes of drugs. As a result, under the PCA unsupervised mode, the urine of gentamicin administration (Figure 4A) on different days was significantly aggregated into six groups, which were 0 d, 1 d, 3 d, 5 d, 7–9 d and 14 d, respectively. The PCA score plot showed that the proportion of the first two components as data total variance was 64.2% and 27.9%. In order to further estimate the sensitivity and accuracy of the recognition pattern, linear discriminant analysis (LDA) was selected to divide the gentamicin-administered group into six clusters according to the urine differences in different degrees of injury (Figure 4B). Moreover, leave-one-out cross-validation was performed to verify the robustness of LDA, which appeared to have a 97.3% between-group accuracy, indicating that the trained classifier could be regarded as a reliable and promising statistical tool. Similarly, the neomycin-treated group was analyzed by PCA, as shown in Figure 4C. The 0 d, 1 d, 3 d, 5–9 d, and 14 d were obviously separated, showing that the first two components accounted for 48.2% and 36.5% of data total variance. And, the same trend was also found in LDA with 97.3% accuracy (Figure 4D). Finally, the tobramycin group was successfully divided into seven groups, the LDA cross-validation accuracy was 97.3% (Figure 4E–F). Taken together, these results indicated that the sensor could be used to quickly identify the progression of kidney injury induced by AGs drugs.

**Figure 5:**
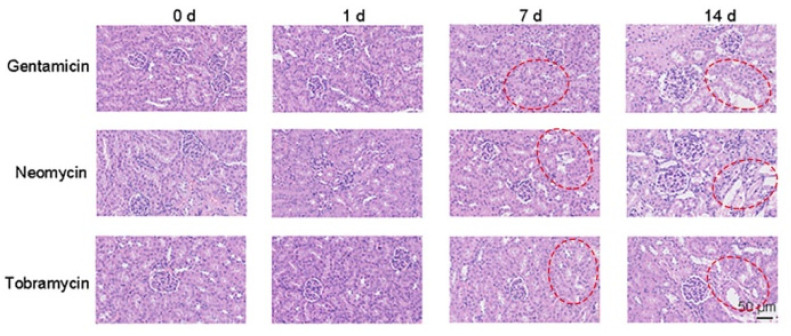
The HE staining imaging of AGs-induced kidney injury model. Scale bars: 50 μm (Location of renal injury: Highlighted in red in the Figure 4 in the manuscript).

Next, the histopathological changes during the development of AGS nephropathy were also obvious by HE staining (Figure 5). Compared with the control group, there was no obvious pathological change after 1 day of administration. All the three drugs showed mild damage of renal tubular epithelial cells from day 7 with a small amount of vacuolar degeneration. On day 14, the damage was more obvious than that on day 7, with a large number of tubular epithelial cells displaying swelling, shedding and vacuolar degeneration.

### 3.4. Discrimination and Evaluation of the Process of PPI Nephropathy Model

**Figure 6:**
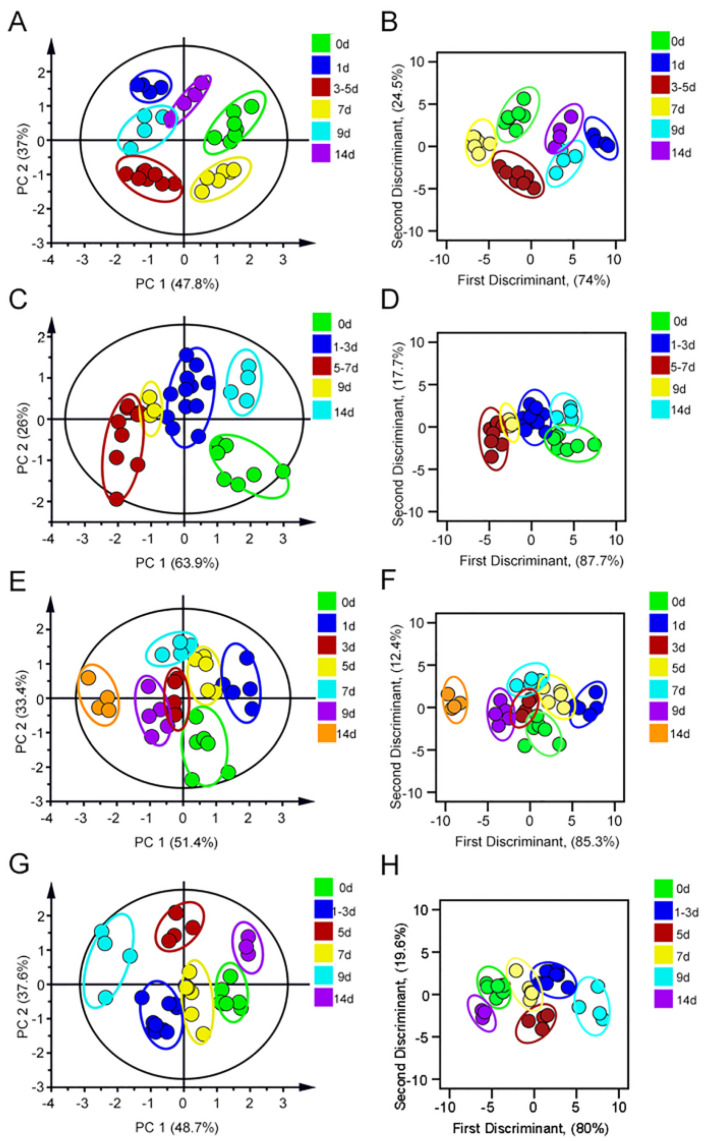
(**A**) The PCA score plot and (**B**) LDA score of Omeprazole-induced kidney injury. (**C**) The PCA score plot and (**D**) LDA score of Lansoprazole-induced kidney injury. (**E**) The PCA score of Pantoprazole-induced kidney injury. (**F**) The LDA score of Pantoprazole-induced kidney injury. (**G**) The PCA score plot of Esomeprazole-induced kidney injury. (**H**) The LDA score of Esomeprazole-induced kidney injury.

**Figure 7:**
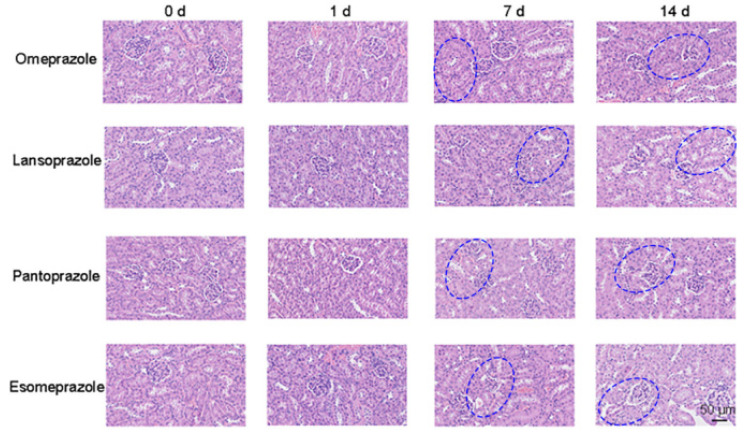
The HE staining imaging of PPI-induced kidney injury model. Scale bars: 50 μm (Location of renal injury: Highlighted in blue in the Figure 6 in the manuscript).

In order to discriminate the damage progression of PPI nephropathy, omeprazole, lansoprazole, pantoprazole, and esomeprazole were selected as the model drugs. The fluorescence intensity of three FLPs in PPIs-induced kidney injury is shown in Table S3. According to PCA results of the omeprazole-treated group, due to the differences in proteinuria (particle size, charge, content, etc.) at different injury stages, this group could be divided into six groups including 0 d, 1 d, 3–5 d, 7 d, 9 d, and 14 d, respectively. The first two principal components accounted for 47.8% and 37% of the data total variance, and the cross-validation suggested 100% accuracy (Figure 6A,B). After PCA and LDA analysis, urine samples of lansoprazole-treated mice were successfully divided into five groups (0 d, 1–3 d, 5–7 d, 9 d, and 14 d) (Figure 6C,D). The PCA plot revealed that pantoprazole-induced renal injury was well isolated (Figure 6E). Moreover, the same grouping result was also showed in the LDA plot with 97.0% accuracy (Figure 6F). Finally, the esomeprazole model was divided into six groups (0 d, 1–3 d, 5 d, 7 d, 9 d, and 14 d), representing six stages of the progression of renal injury. Then, LDA was used to verify again, and the accuracy was 97.1% (Figure 6G,H). Moreover, the histopathological changes with the development of PPI nephropathy were also explored. As shown in Figure 7, there was mild hemorrhage and necrosis on day 7. There was also mild damage of renal tubular epithelial cells, and the damage was more pronounced on day 14 than on day 7.

### 3.5. Discrimination and Evaluation of the Process of NSAIDs Nephropathy Model

**Figure 8:**
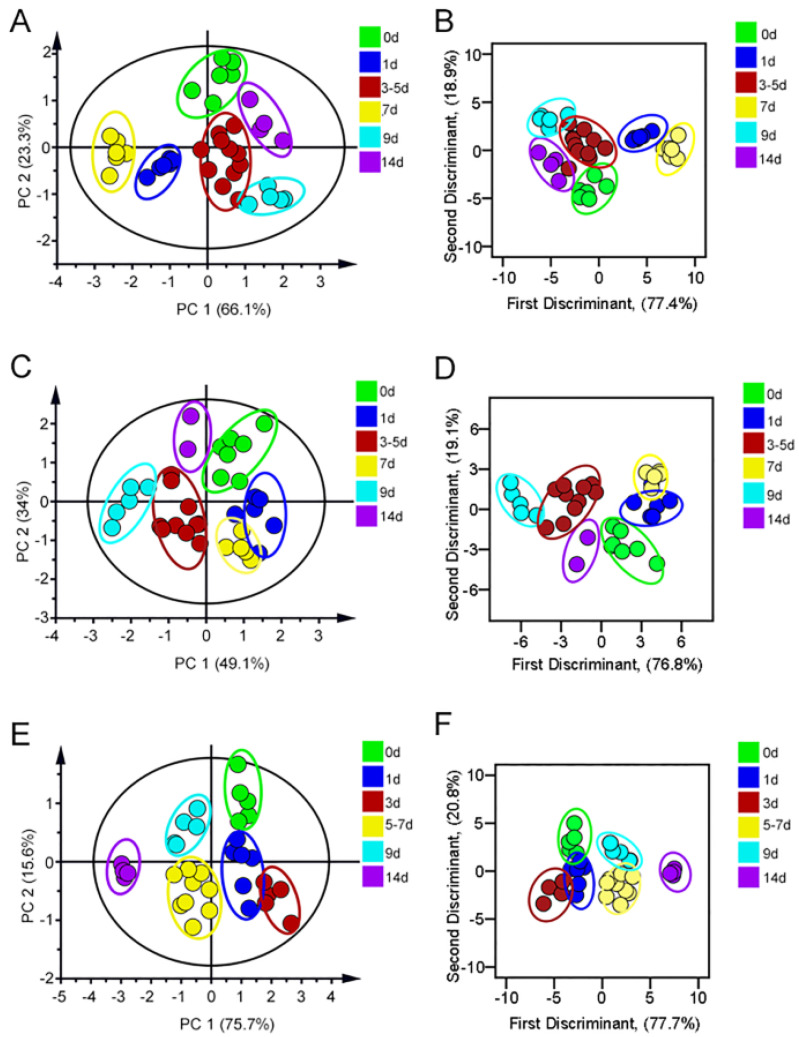
(**A**) The PCA score plot and (**B**) LDA score of Ibuprofen-induced kidney injury. (**C**) The PCA score of Diclofenac-induced kidney injury. (**D**) The LDA score of Diclofenac-induced kidney injury. (**E**) The PCA score plot and (**F**) LDA score of Naproxen-induced kidney injury.

PCA and LDA were used to identify and analyze the progression of NSAIDs kidney injury. The results showed that ibuprofen-treated group was divided into six groups, which were 0 d, 1 d, 3–5 d, 7 d, 9 d and 14 d, respectively. The fluorescence intensity of three FLPs in NSAIDs-induced kidney injury is shown in Table S4. The LDA cross-validation accuracy rate reached 97.4% (Figure 8A,B). In addition, the diclofenac group appeared to have the same trend as ibuprofen, and the LDA cross-validation accuracy was 100% (Figure 8C,D). Meanwhile, the naproxen administration group was divided into six groups (0 d, 1 d, 3 d, 5–7 d, 9 d and 14 d). The first two principal components accounted for 75.7% and 15.6% of the data total variance with a cross-validation accuracy of 94.3% (Figure 8E,F).

**Figure 9:**
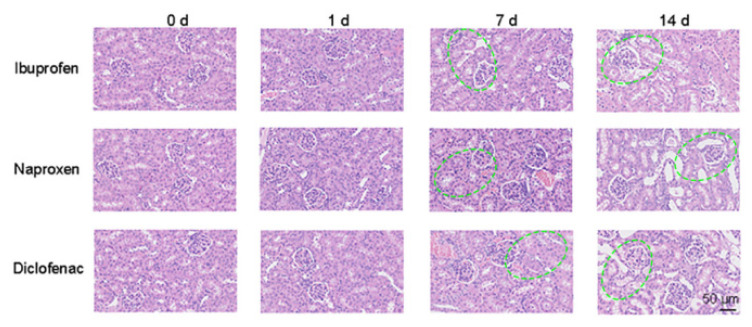
The HE staining imaging of NSAIDs-induced kidney injury model. Scale bars: 50 μm (Location of renal injury: Highlighted in green in the Figure 8 in the manuscript).

HE staining was used to analyze the histopathological changes in NSAIDS nephropathy at different stages of injury (Figure 9). The damage area was increased significantly with the prolongation of the administration time. The renal tubular epithelial cells were swollen, degenerated, necrotic, and vacuolated. Meanwhile a large number of inflammatory cells were infiltrated with glomerular degeneration and necrosis.

### 3.6. Discrimination and Evaluation of the Different Types of DIKI

**Figure 10:**
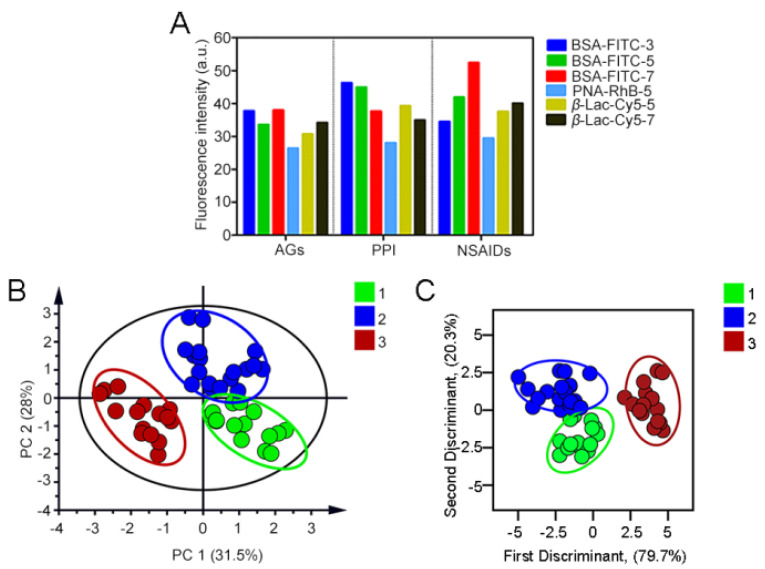
(**A**) Fluorescence response patterns generated by AuNPs–FLPs sensor array for identifying different kinds of DIKIs. Two-dimensional PCA score plot (**B**) and LDA plot (**C**) for discrimination and evaluation of the different types of DIKIs according to the VIP value used. 1: aminoglycoside antibiotics-induced renal injury; 2: proton pump inhibitors-induced renal injury; 3: non-steroidal anti-inflammatory drugs-induced renal injury.

Based on the development characteristics of proteinuria of different DIKI types and the time–effect relationship of DIKI, AuNPs–PEI/FLPs sensors were applied in the mice urine of different types of DIKI at different time points. In order to initially identify the action categories of the three DIKIs, we employed PCA to distinguish the fluorescence response pattern of diverse types of DIKI (Figure S19). The results showed that the three DIKIs (AGs, PPI, and NSAIDs) could be separated, but there still existed a lot of overlap. Subsequently, we further analyzed the results by using the VIP value (variable importance for the projection, which summarized the importance of the variables both to explain X and to correlate to Y, >1) (Table S5). As can be seen from Figure 10A, each type of DIKI formed a unique fluorescent fingerprint, and the PCA plot (Figure 10B) revealed that the three types of DIKI model were obviously separated. The first two principal components accounted for 31.5% and 28.0% of the data total variance. Meanwhile, LDA was also applied to divide the 49 data points into 3 obvious clusters in terms of the proteinuria differences in different DIKI types (Figure 10C). The three discriminant functions of DIKI types were as follows:

Y1 = 1.186X1 − 0.353X2 + 7.049X3 + 1.514X4 + 2.491X5 + 0.909X6 − 177.657;

Y2 = 0.411X1 + 0.017X2 + 7.264X3 + 2.039X4 + 2.210X5 + 0.615X6 − 205.042;

Y3 = −0.556X1 − 0.191X2 + 7.916X3 + 1.965X4 + 3.477X5 + 1.440X6 − 260.784.

where Y1 denotes the AGs-induced renal injury: dose-dependent toxic reaction, which causes tubular cytotoxic reaction by damaging mitochondrial or lysosomal function, and leads to tubular epithelial cell necrosis; Y2 denotes the PPI-induced renal injury: non-dose-dependent toxic reactions, which mainly relates to inflammation; and Y3 denotes the NSAIDs-induced renal injury: multiple DIKI manifestations, hemodynamic changes resulting in renal ischemic injury or nephrotic range proteinuria from glomerular injury. X1–X6 indicate the fluorescence intensity of the six signal channels, respectively. Furthermore, the leave-one-out cross-validation suggested 100% between-group accuracy. And, the training matrix of fluorescence response patterns of the sensor to the three DIKI types is shown in Table S6.

## 4. Conclusions

In summary, we successfully fabricated a multichannel fluorescence array sensor composed of non-covalent interaction of AuNPs–PEI and three FLPs, which exhibited a series of advantages including simple preparation, mild reaction conditions, high stability, rapid response, low cost, and easy batch preparation. The sensor was successfully applied to identify and evaluate the progression of DIKI via PCA and LDA based on the differences in protein type and content in urine at different stages of kidney injury. Furthermore, the time–effect relationship of DIKI was introduced to increase information content and greatly expand the number of signal channels, utilizing this benefit to distinguish proteinuria altered by the three types of DIKIs (AGS, PPI, and NASIDs). It was worth noting that this sensor has a wide range of applications and can be used to identify the development of renal disease and explore the specific mechanism of nephrotoxic drugs, which also provides a promising approach for screening potential renal-protective drugs.

## Data Availability

The data presented in this study are available on request.

## Supplementary Materials

The following supporting information can be downloaded at: https://www.mdpi.com/article/10.3390/s23136114/s1, Figure S1: TEM image of AuNPs, Figure S2: Size characterization of AuNPs, Figure S3: Size characterization of AuNPs–PEI/FLPs nanosensor, Figure S4: Zeta potential characterization of AuNPs, Figure S5: Zeta potential characterization of AuNPs–PEI. Figure S6: Zeta potential characterization of AuNPs–PEI/FLPs nanosensor, Figure S7: Hydrodynamic diameter of AuNPs–PEI over a period of 2 weeks, Figure S8: Zeta potential of AuNPs–PEI over a period of 2 weeks, Figure S9: Excitation and emission fluorescence spectra of BSA–FITC, Figure S10: Excitation and emission fluorescence spectra of PNA–RhB, Figure S11: Excitation and emission fluorescence spectra of β–Lac–Cy5, Figure S12: Normalized fluorescence intensity of FLPs incubation with AuNPs–PEI for different times, Figure S13: Effects of pH (from 4.5 to 8.5) on BSA–FITC and AuNPs–PEI, Figure S14: Effects of pH (from 4.5 to 8.5) on PNA–RhB and AuNPs–PEI, Figure S15: Effects of pH (from 4.5 to 8.5) on β–Lac–Cy5 and AuNPs–PEI, Figure S16: Effects of inorganic ions on BSA–FITC and AuNPs–PEI. MMP–2: matrix metalloproteinase–2. CysC: Cystatin C. GSH: glutathione. ALB: albumin. Figure S17: Effects of inorganic ions on PNA–RhB and AuNPs–PEI. MMP–2: matrix metalloproteinase–2. CysC: Cystatin C. GSH: glutathione. ALB: albumin, Figure S18: Effects of inorganic ions on β–Lac–Cy5 and AuNPs–PEI. MMP–2: matrix metalloproteinase–2. CysC: Cystatin C. GSH: glutathione. ALB: albumin, Figure S19: Two-dimensional PCA score plot for discrimination and evaluation of the different types of DIKI; Table S1: Binding parameters for AuNPs–PEI and FLPs as determined by the fitting of the fluorescence titration curves, Table S2. The fluorescence intensity of three FLPs in AGs-induced kidney injury (*n* = 5), Table S3. The fluorescence intensity of three FLPs in PPIs-induced kidney injury (*n* = 5), Table S4. The fluorescence intensity of three FLPs in NSAIDs-induced kidney injury (*n* = 5), Table S5: The VIP value for identifying the three types of DIKI (3, 5, and 7 represent days of administration), Table S6: The training matrix of fluorescence response patterns of AuNPs–PEI/FLPs sensor against the three DIKI types.
