# Supplementary material for: A Multichannel Fluorescent Array Sensor for Discrimination of Different Types of Drug-Induced Kidney Injury
Source: Sensors (Basel). 2023 Jul 3;23(13):6114. doi: 10.3390/s23136114 (PMC10346614; doi:10.3390/s23136114)
Supplement: Supplementary file 1 [file sensors-23-06114-s001.zip › sensors-2409023-supplementary.pdf]

A Multichannel Fluorescent Array Sensor for Discrimination of Different Types of Drug–Induced Kidney Injury

Kunhui Sun [1,2,†], Bing Wang [2,†], Jiaoli Lin [1], Lei Han [1], Meifang Li [2], Ping Wang [2], Xie-an Yu [2,*] and Jiangwei Tian [1,*]

[1] State Key Laboratory of Natural Medicines, Jiangsu Key Laboratory of TCM Evaluation and Translational Research, School of Traditional Chinese Pharmacy, China Pharmaceutical University, Nanjing 211198, China

[2] NMPA Key Laboratory for Bioequivalence Research of Generic Drug Evaluation, Shenzhen Institute for Drug Control, Shenzhen 518057, China

† These authors contributed equally to this work.

* Corresponding authors: yuxieanalj@126.com (Y.X.); jwtian@cpu.edu.cn (J.T.)

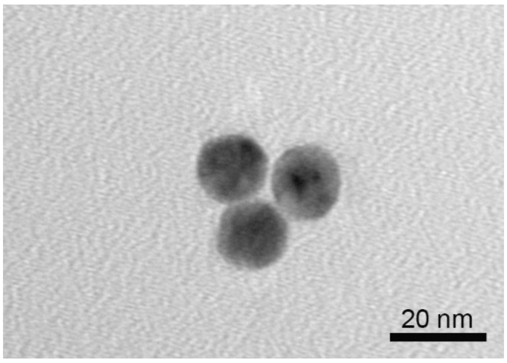

Figure S1. TEM image of AuNPs.

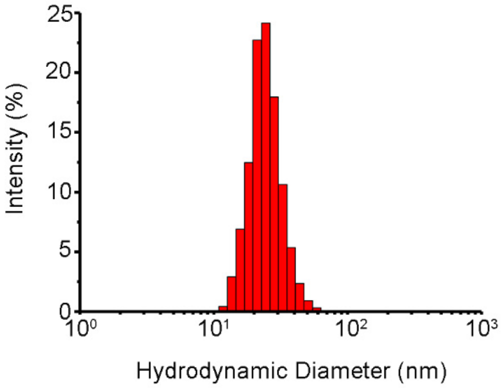

Figure S2. Size characterization of AuNPs.

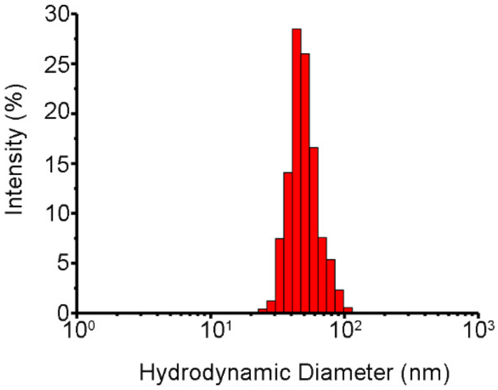

Figure S3. Size characterization of AuNPs–PEI/FLPs nanosensor.

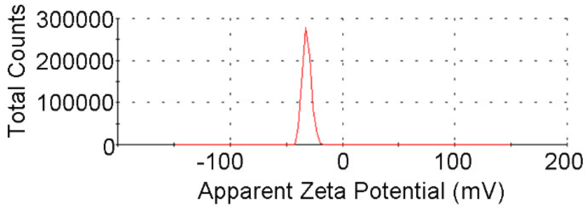

Figure S4. Zeta potential characterization of AuNPs.

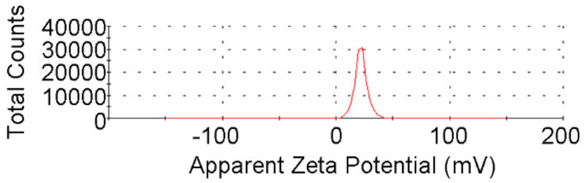

Figure S5. Zeta potential characterization of AuNPs–PEI.

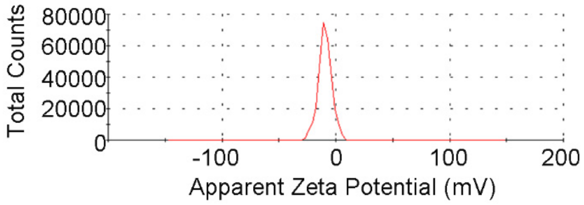

Figure S6. Zeta potential characterization of AuNPs–PEI/FLPs nanosensor.

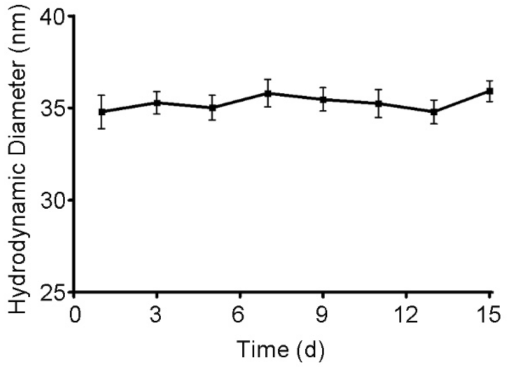

Figure S7. Hydrodynamic diameter of AuNPs–PEI during 2 weeks.

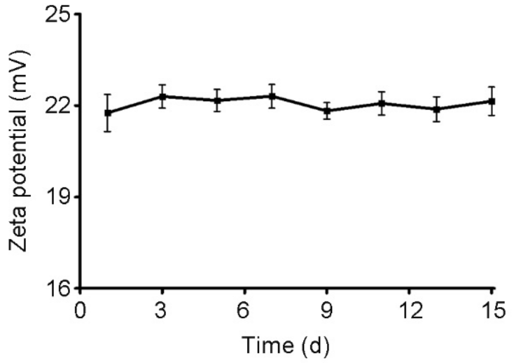

Figure S8. Zeta potential of AuNPs–PEI during 2 weeks.

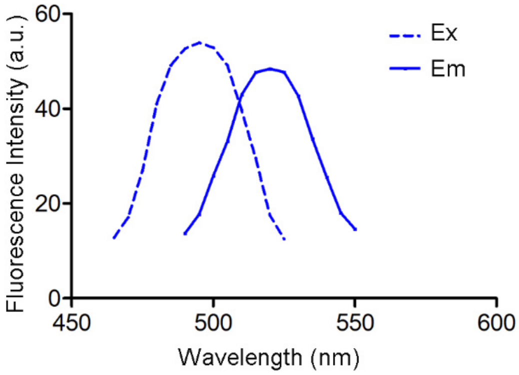

Figure S9. Excitation and emission fluorescence spectra of BSA–FITC.

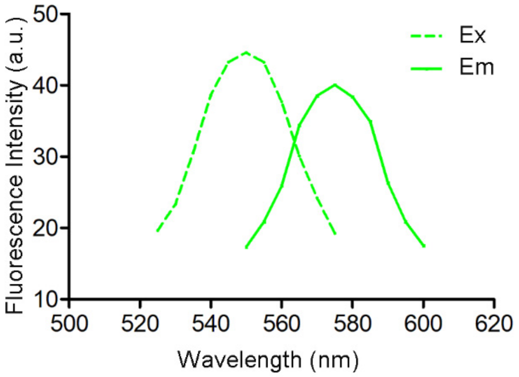

Figure S10. Excitation and emission fluorescence spectra of PNA–RhB.

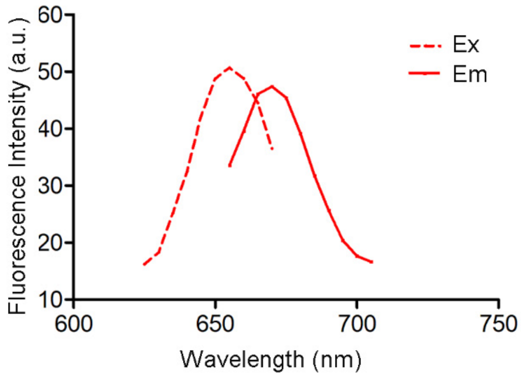

Figure S11. Excitation and emission fluorescence spectra of β–Lac–Cy5.

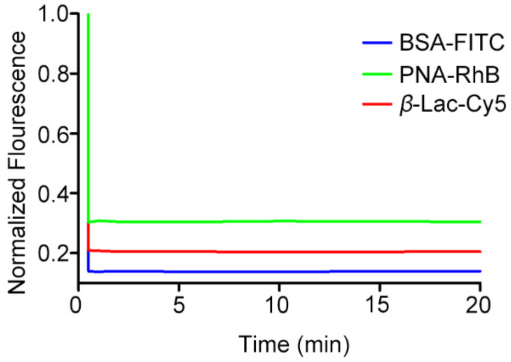

Figure S12. Normalized fluorescence intensity of FLPs incubation with AuNPs–PEI for different times.

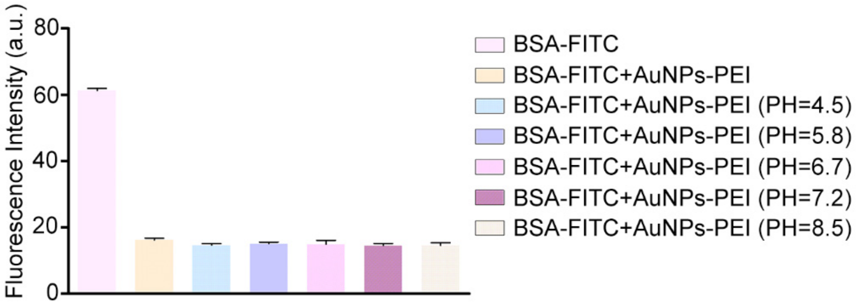

Figure S13. Effects of pH (from 4.5 to 8.5) on BSA–FITC and AuNPs–PEI.

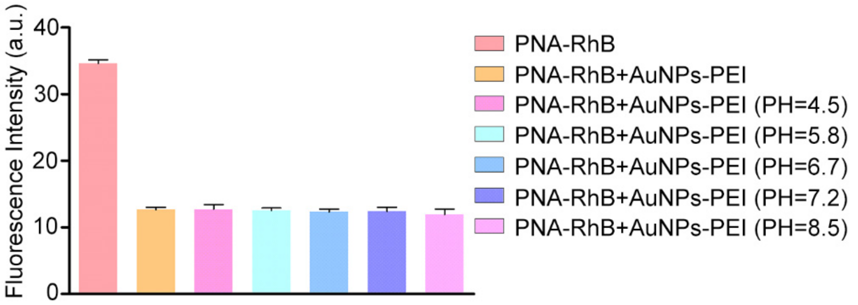

Figure S14. Effects of pH (from 4.5 to 8.5) on PNA–RhB and AuNPs–PEI.

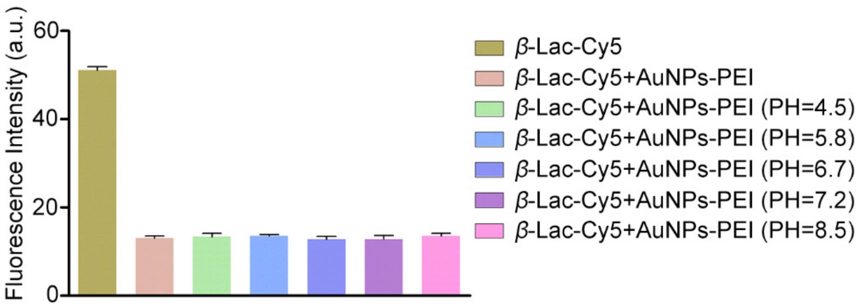

Figure S15. Effects of pH (from 4.5 to 8.5) on β–Lac–Cy5 and AuNPs–PEI.

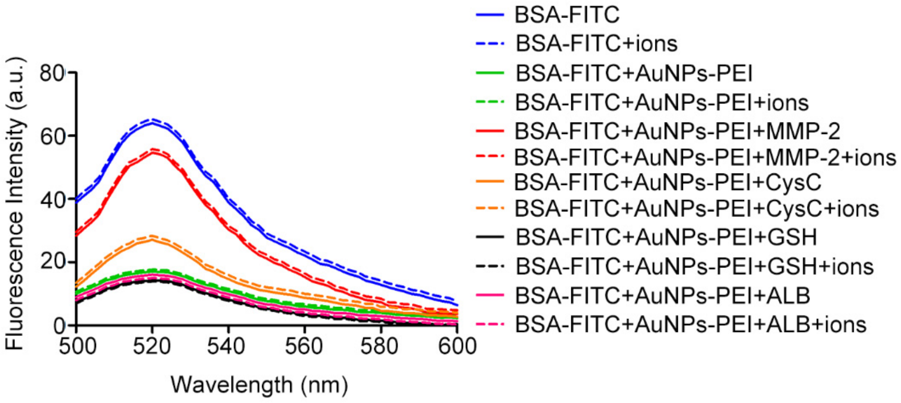

Figure S16. Effects of inorganic ions on BSA–FITC and AuNPs–PEI. MMP–2: matrix metalloproteinase–2. CysC: Cystatin C. GSH: glutathione. ALB: albumin.

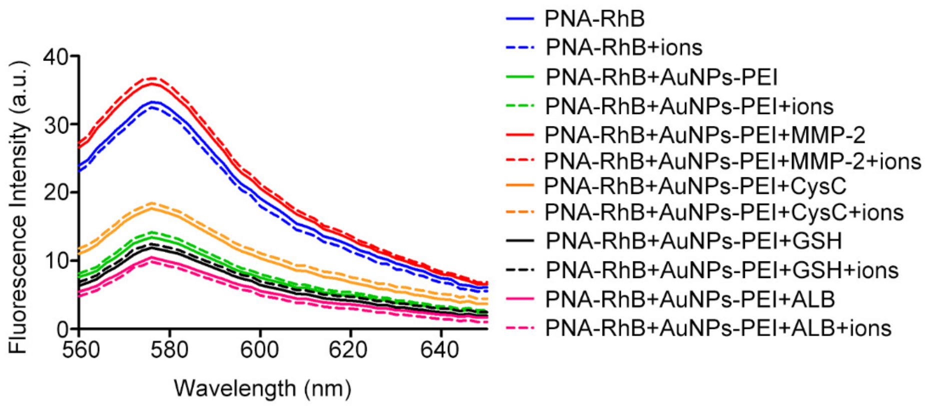

Figure S17. Effects of inorganic ions on PNA–RhB and AuNPs–PEI. MMP–2: matrix metalloproteinase–2. CysC: Cystatin C. GSH: glutathione. ALB: albumin.

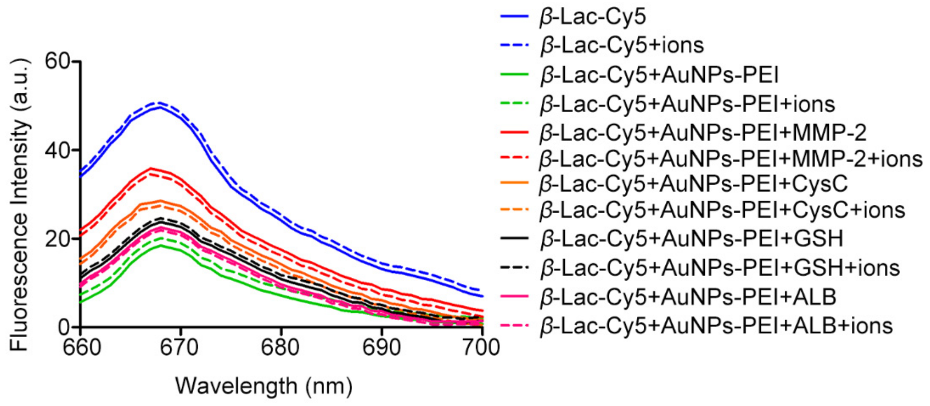

Figure S18. Effects of inorganic ions on β–Lac–Cy5 and AuNPs–PEI. MMP–2: matrix metalloproteinase–2. CysC: Cystatin C. GSH: glutathione. ALB: albumin.

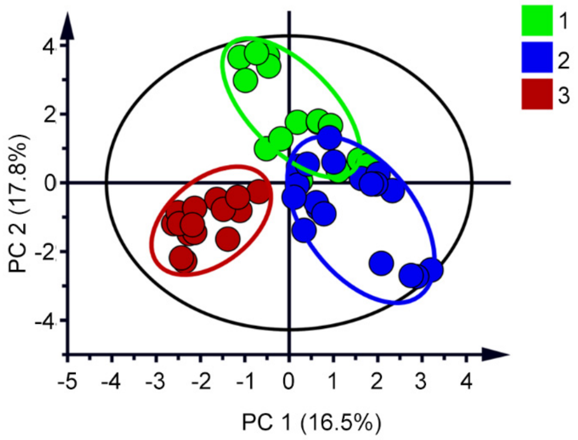

Figure S19. Two dimensional PCA score plot for discrimination and evaluation the different types of DIKI.

Table S1. Binding parameters for AuNPs–PEI and FLPs as determined by the fitting of the fluorescence titration curves

Fluorophore	Binding constant (K_a), (M^{-1})	R^2
BSA–FITC	1.41×10^{8}	0.999
PNA–RhB	2.68×10^{6}	0.993
β–Lac–Cy5	7.43×10^{7}	0.998

Table S2. The fluorescence intensity of three FLPs in AGs-induced kidney injury (n=5).

Drug name	Number	BSA-FITC	PNA-RhB	β-Lac-Cy5
	0-1	42.29	29.36	26.65
	0-2	40.91	30.86	28.22
	0-3	43.29	29.58	30.45
	0-4	43.92	29.52	25.24
	0-5	48.14	31.16	22.75
	0-6	40.91	32.74	32.11
	0-7	46.42	30.00	25.45
	1-1	47.51	32.96	29.18
	1-2	47.40	32.02	32.60
	1-3	52.54	32.64	29.68
	1-4	56.70	35.51	29.88
	1-5	51.04	33.28	30.34
	3-1	39.53	29.30	33.44
	3-2	40.38	28.29	32.66
	3-3	44.10	29.42	32.25
	3-4	37.81	29.13	34.89
	3-5	41.24	29.04	32.68
	3-6	47.55	28.24	32.46
	5-1	31.62	24.18	32.57
	5-2	31.19	26.83	37.05
Gentamicin	5-3	27.06	26.01	34.68
	5-4	31.50	25.96	32.34
	5-5	32.30	26.38	33.94
	7-1	33.85	26.43	41.70
	7-2	40.59	25.57	39.71
	7-3	33.53	24.95	38.31
	7-4	32.62	24.20	41.98
	7-5	43.32	24.51	38.53
	7-6	43.91	29.12	39.61
	9-1	46.55	25.42	36.57
	9-2	43.85	23.60	36.28
	9-3	45.28	25.14	36.92
	9-4	45.23	24.72	36.59
	14-1	53.80	33.78	36.42
	14-2	58.52	33.47	38.50
	14-3	56.16	33.63	37.46
	14-4	55.16	33.46	36.23
	0-1	42.29	29.36	26.65
	0-2	40.91	30.86	28.22
	0-3	43.29	29.58	30.45
	0-4	43.92	29.52	25.24
	0-5	48.14	31.16	22.75
	0-6	40.91	32.74	32.11
	0-7	46.42	30.00	25.45
	1-1	48.48	28.60	34.31
	1-2	44.33	31.68	34.50

	1-3	43.29	32.07	37.08
	1-4	43.49	31.15	37.99
	3-1	32.59	21.93	32.38
	3-2	43.33	21.80	34.87
	3-3	37.57	21.86	35.16
	3-4	31.91	23.10	33.78
	3-5	33.72	23.02	36.68
	5-1	31.49	25.84	26.20
	5-2	32.50	26.22	23.33
Neomycin	5-3	36.61	25.09	24.45
	5-4	36.88	25.50	21.17
	5-5	39.03	27.26	22.73
	7-1	41.48	26.71	27.20
	7-2	41.09	27.06	26.70
	7-3	40.02	28.52	27.20
	7-4	39.19	26.59	28.47
	7-5	37.41	27.03	26.43
	9-1	27.64	29.48	24.90
	9-2	27.94	28.60	25.72
	9-3	26.02	27.55	26.48
	9-4	27.73	25.76	27.59
	9-5	27.33	27.85	26.17
	14-1	42.58	27.54	40.09
	14-2	42.80	26.72	38.90
	14-3	42.58	27.23	39.50
	0-1	42.29	29.36	26.65
	0-2	42.19	25.79	26.22
	0-3	30.63	30.00	20.84
	0-4	40.91	30.86	28.22
	0-5	43.29	29.58	30.45
	0-6	43.92	29.52	25.24
	0-7	46.42	30.00	25.45
	0-8	36.70	25.95	29.56
	1-1	54.30	32.36	40.79
	1-2	55.54	29.41	37.88
	1-3	55.38	33.22	40.72
	1-4	55.18	31.24	37.42
	1-5	53.68	30.89	40.17
	3-1	36.42	33.11	41.33
	3-2	36.35	30.10	38.27
	3-3	36.97	29.13	38.78
	3-4	39.16	30.49	41.41
	3-5	34.32	32.33	41.23
	5-1	37.56	28.48	33.79
	5-2	33.42	27.70	34.46
Tobramycin	5-3	36.88	27.09	36.30
	5-4	38.01	26.75	34.32
	5-6	37.62	28.18	34.34
	7-1	38.59	33.23	35.99

7-2	37.46	35.36	38.20
7-3	35.75	32.50	33.68
7-4	37.28	33.16	32.58
7-5	37.43	31.81	35.38
9-1	48.37	33.05	43.09
9-2	50.79	31.76	44.89
9-3	45.28	33.02	45.03
9-4	51.48	34.14	43.61
9-5	40.82	34.70	40.33
14-1	32.72	24.22	38.23
14-2	31.65	25.06	37.12
14-3	32.19	24.64	37.67
14-4	30.90	24.67	38.09

Table S3. The fluorescence intensity of three FLPs in PPIs-induced kidney injury (n=5).

Drug name	Number	BSA-FITC	PNA-RhB	β-Lac-Cy5
	0-1	42.29	29.36	26.65
	0-2	40.91	30.86	28.22
	0-3	43.29	29.58	30.45
	0-4	43.92	29.52	25.24
	0-5	48.14	31.16	22.75
	0-6	40.91	32.74	32.11
	0-7	46.42	30.00	25.45
	1-1	64.74	29.20	35.34
	1-2	65.64	28.56	36.62
	1-3	66.39	29.12	39.08
	1-4	66.89	29.03	39.88
	3-1	39.13	26.29	35.89
	3-2	44.87	26.01	44.96
	3-3	44.43	26.21	41.39
Omeprazole	5-1	39.64	26.75	42.98
	5-2	40.69	25.89	37.58
	5-3	44.31	26.42	41.36
	5-4	43.33	26.16	38.84
	5-5	42.99	26.02	42.67
	7-1	31.28	29.24	30.15
	7-2	30.41	29.64	32.68
	7-3	29.15	30.04	31.48
	7-4	31.01	28.54	31.93
	7-5	42.19	25.79	26.22
	7-6	36.70	25.95	29.56
	9-1	51.09	29.86	40.60
	9-2	54.07	26.01	40.75
	9-3	56.72	28.58	40.98
	9-4	55.19	27.50	39.54
	14-1	48.93	30.29	40.10
	14-2	48.21	32.71	38.94
	14-3	49.10	31.70	39.41
	14-4	56.28	31.70	32.86
	0-1	42.29	29.36	26.65
	0-2	48.48	28.60	34.31
	0-3	40.91	30.86	28.22
	0-4	43.29	29.58	30.45
	0-5	43.92	29.52	25.24
	0-6	48.14	31.16	22.75
	0-7	46.42	30.00	25.45
	1-1	39.65	32.17	35.27
	1-2	38.72	31.43	36.83
	1-3	41.04	30.86	39.57
	1-4	46.28	28.33	36.49
	1-5	40.05	30.17	36.72
	1-6	39.77	30.85	35.07

	3-1	39.19	30.02	39.37
	3-2	39.65	31.90	40.73
	3-3	41.27	28.57	39.28
	3-4	39.57	28.95	36.49
	3-5	38.68	31.06	37.35
	3-6	39.98	32.02	38.71
	5-1	32.84	28.07	40.31
Lansoprazole	5-2	36.02	28.12	42.35
	5-3	35.36	28.79	39.75
	5-4	36.19	28.19	42.51
	5-5	36.14	28.41	40.39
	7-1	32.14	25.76	33.60
	7-2	32.23	27.89	33.52
	7-3	33.88	27.56	39.44
	7-4	33.98	26.55	38.38
	9-1	36.89	29.16	39.00
	9-2	36.19	29.58	40.11
	9-3	36.12	29.09	38.48
	9-4	36.51	29.31	38.63
	14-1	46.81	32.95	35.66
	14-2	47.67	31.59	33.52
	14-3	46.99	32.56	34.88
	14-4	40.91	32.74	32.11
	0-1	43.84	31.07	27.51
	0-2	42.10	32.42	27.88
	0-3	43.49	31.25	29.92
	0-4	43.61	30.49	23.88
	0-5	50.15	32.62	24.43
	0-6	46.99	31.39	27.30
	1-1	58.56	30.03	32.67
	1-2	56.06	31.76	32.01
	1-3	60.57	32.36	32.22
	1-4	53.99	34.34	30.48
	1-5	57.59	32.95	35.28
	3-1	46.20	28.14	30.41
	3-2	47.79	27.73	31.18
	3-3	47.49	28.16	33.54
	3-4	47.86	27.85	33.17
	3-5	45.06	28.54	30.07
	5-1	54.78	29.13	35.42
	5-2	54.47	30.27	32.05
Pantoprazole	5-3	41.39	32.03	35.60
	5-4	54.50	29.42	34.74
	5-5	41.60	33.16	32.58
	7-1	39.91	31.99	36.97
	7-2	39.75	28.92	36.01
	7-3	38.62	31.24	36.23
	7-4	38.26	31.38	37.28
	9-1	38.54	27.66	32.94

	9-2	37.94	28.97	30.79
	9-3	37.48	29.85	29.22
	9-4	42.34	26.69	27.98
	9-5	37.07	28.01	30.11
	14-1	30.00	23.60	33.95
	14-2	30.47	24.46	31.06
	14-3	30.14	25.53	32.09
	14-4	23.46	27.55	31.07
Esomeprazole	0-1	41.59	30.62	24.25
	0-2	40.00	29.09	30.90
	0-3	43.82	30.04	29.70
	0-4	44.07	31.11	28.23
	0-5	46.32	29.41	22.40
	0-6	40.24	31.72	30.92
	0-7	48.06	30.07	24.48
	1-1	55.50	35.13	34.14
	1-2	56.00	30.04	32.20
	1-3	59.16	35.05	34.82
	1-4	57.58	32.11	31.60
	1-5	60.28	34.99	33.19
	1-6	60.78	34.31	34.37
	3-1	65.15	32.41	37.14
	3-2	47.05	33.80	38.75
	3-3	60.97	34.08	29.92
	3-4	62.24	34.06	32.79
	3-5	48.56	33.91	39.47
	5-1	53.24	27.40	41.44
	5-2	55.94	25.02	41.63
	5-3	55.02	27.82	41.32
	5-4	55.05	25.16	41.89
	5-5	44.79	26.99	42.35
	7-1	35.35	33.80	42.47
	7-2	50.42	31.08	34.89
	7-3	48.47	30.04	36.99
	7-4	55.63	30.25	32.19
	7-5	51.02	33.48	26.01
	9-1	62.64	30.32	45.55
	9-2	70.78	33.88	43.28
	9-3	74.04	28.39	48.42
	9-4	72.38	29.37	47.47
	14-1	42.78	25.67	26.67
	14-2	36.86	25.33	31.74
	14-3	30.99	27.26	34.86

Table S4. The fluorescence intensity of three FLPs in NSAIDs-induced kidney injury (n=5).

Drug name	Number	BSA-FITC	PNA-RhB	β-Lac-Cy5
	0-1	42.29	29.36	26.65
	0-2	40.91	30.86	28.22
	0-3	43.29	29.58	30.45
	0-4	43.92	29.52	25.24
	0-5	40.91	32.74	32.11
	0-6	46.42	30.00	25.45
	1-1	53.34	28.19	37.25
	1-2	56.44	27.63	36.17
	1-3	55.51	29.07	40.00
	1-4	54.53	28.63	38.22
	1-5	52.70	28.35	36.90
	1-6	54.50	28.37	37.71
	3-1	31.17	27.40	36.27
	3-2	28.07	30.48	33.33
	3-3	36.97	25.77	33.80
	3-4	34.58	26.97	37.73
	3-5	35.79	28.71	37.57
	3-6	29.12	26.25	38.29
Ibuprofen	5-1	44.61	28.12	34.85
	5-2	38.09	26.17	32.95
	5-3	40.80	28.18	32.71
	5-4	35.11	27.95	34.01
	5-5	40.67	27.69	33.06
	7-1	53.18	32.77	40.75
	7-2	52.22	33.78	42.22
	7-3	56.31	32.43	43.47
	7-4	54.07	33.97	40.61
	7-5	57.10	34.10	40.25
	7-6	54.58	33.41	41.46
	9-1	36.51	23.11	36.04
	9-2	33.52	20.86	32.79
	9-3	28.90	22.38	34.16
	9-4	29.19	24.20	34.31
	9-5	32.03	22.64	34.33
	14-1	33.77	26.33	29.81
	14-2	31.24	24.39	28.97
	14-3	30.48	29.59	29.25
	14-4	31.83	26.77	29.34
	0-1	42.29	29.36	26.65
	0-2	40.91	30.86	28.22
	0-3	43.92	29.52	25.24
	0-4	48.14	31.16	22.75
	0-5	56.28	31.70	32.86
	0-6	46.42	30.00	25.45
	1-1	43.64	26.27	31.07
	1-2	42.47	29.57	32.36

	1-3	38.41	29.81	31.34
	1-4	42.22	29.74	30.51
	1-5	38.56	26.12	36.43
	1-6	40.09	27.40	34.67
	3-1	32.70	26.27	32.26
	3-3	31.46	25.18	30.13
	3-4	29.44	22.81	32.42
	3-5	32.89	26.12	33.37
	3-6	39.71	24.89	31.94
	5-1	46.94	32.69	44.44
Naproxen	5-2	49.29	31.36	43.82
	5-3	40.65	34.41	39.99
	5-4	40.20	33.91	38.04
	7-1	51.86	31.63	40.12
	7-2	52.12	33.30	42.36
	7-3	41.26	32.09	44.94
	7-4	39.99	31.05	41.19
	7-5	52.56	27.40	40.07
	9-1	56.67	32.95	39.21
	9-2	57.52	32.46	39.32
	9-4	50.49	35.05	35.40
	9-5	58.17	30.21	35.14
	9-6	56.28	31.70	32.86
	14-1	66.26	34.87	49.57
	14-2	67.43	35.48	48.51
	14-3	66.84	35.18	49.04
	14-4	65.54	34.87	48.65
	0-1	42.29	29.36	26.65
	0-2	40.91	30.86	28.22
	0-3	43.29	29.58	30.45
	0-4	43.92	29.52	25.24
	0-5	48.14	31.16	22.75
	0-6	40.91	32.74	32.11
	0-7	46.42	30.00	25.45
	1-1	51.92	30.31	32.91
	1-2	48.85	31.42	32.24
	1-3	47.99	29.97	34.39
	1-4	48.66	33.17	39.99
	1-5	48.53	32.12	32.75
	1-6	51.55	32.98	35.64
	3-1	35.59	31.53	38.60
	3-2	37.06	29.35	35.57
	3-3	37.19	26.68	30.76
	3-4	37.20	28.31	37.76
	3-5	37.49	26.45	37.48
	3-6	30.01	29.92	33.75
	5-1	40.97	29.75	39.68
Diclofenac	5-2	40.31	25.61	37.10
	5-3	43.45	27.92	36.32

5-4	41.58	27.76	37.70
7-1	54.40	29.96	39.03
7-2	55.93	28.60	37.35
7-3	53.50	28.74	35.15
7-4	55.98	26.34	36.28
7-5	54.95	28.41	36.95
9-1	32.18	23.63	36.13
9-2	33.07	23.09	37.93
9-3	31.70	25.01	35.07
9-4	32.86	26.28	33.29
9-5	31.55	24.95	33.30
14-1	42.19	25.79	26.22
14-2	36.41	27.90	23.53

Table S5. The VIP value of identification the three types of DIKI (3, 5, and 7 represent days of administration)

Var ID (primary)	VIP
BSA–FITC–7	1.69164
BSA–FITC–3	1.40387
β–Lac–Cy5–5	1.38330
BSA–FITC–5	1.31458
β–Lac–Cy5–7	1.19527
PNA–RhB–5	1.05385

Table S6. The training matrix of fluorescence response patterns of AuNPs–PEI/FLPs sensor against the three DIKI types (n=5).

	**Drug name**	**BSA–FITC–3**	**BSA–FITC–5**	**PNA–RhB–5**	**β–Lac–Cy5–5**	**BSA–FITC–7**	**β–Lac–Cy5–7**
	gentamicin	39.5263	31.6187	24.1776	32.5692	33.8467	41.6963
	gentamicin	40.3848	31.1930	26.8298	37.0489	40.5949	39.7112
	gentamicin	44.0951	27.0634	26.0146	34.6765	33.5250	38.3102
	gentamicin	37.8056	31.5034	25.9602	32.3440	32.6238	41.9842
	gentamicin	41.2400	32.2958	26.3772	33.9402	43.3223	38.5322
	neomycin	32.5895	31.4898	25.8412	26.2019	41.4822	27.2006
	neomycin	43.3313	32.5004	26.2237	23.3322	41.0896	26.6977
	neomycin	37.5684	36.6060	25.0939	24.4511	40.0216	27.2038
	neomycin	31.9121	36.8767	25.4975	21.1692	39.1893	28.4742
	neomycin	33.7216	39.0335	27.2585	22.7269	37.4109	26.4321
	tobramycin	36.4247	37.5641	28.4767	33.7944	38.5918	35.9887
	tobramycin	36.3533	33.4189	27.6971	34.4623	37.4603	38.1951
AGs	tobramycin	36.9739	36.8751	27.0912	36.3011	35.7462	33.6776
	tobramycin	39.1583	38.0051	26.7530	34.3198	37.2785	32.5803
	tobramycin	34.3228	27.4164	26.2679	32.7086	37.4347	35.3796
	lansoprazole	39.1910	32.8406	28.0713	40.3098	32.1355	33.6008
	lansoprazole	39.6498	36.0188	28.1210	42.3468	32.2332	33.5196
	lansoprazole	41.2653	35.3564	28.7898	39.7543	33.8825	39.4444
	lansoprazole	39.5669	36.1873	28.1882	42.5129	33.9782	38.3842
	lansoprazole	38.6760	36.1410	28.4111	40.3927	33.0574	36.2372
	omeprazole	39.1264	39.6441	26.7452	42.9824	31.2804	30.1527
	omeprazole	44.8651	40.6936	25.8868	37.5834	30.4091	32.6794
	omeprazole	44.4332	44.3137	26.4208	41.3609	29.1521	31.4768
	omeprazole	42.8082	43.3292	26.1566	38.8355	31.0107	31.9294
	omeprazole	39.8082	42.9908	26.0182	42.6722	42.1922	26.2244
	pantoprazole	46.1981	54.7838	29.1327	35.4156	39.9085	36.9717
	pantoprazole	47.7899	54.4737	30.2712	32.0455	39.7527	36.0066
	pantoprazole	47.4948	41.3863	32.0340	35.5978	38.6191	36.2348
	pantoprazole	47.8569	54.5047	29.4215	34.7418	38.2612	37.2846
	pantoprazole	45.0612	41.5985	33.1601	32.5803	39.1354	36.6244
	esomeprazole	65.1510	53.2432	27.4044	41.4411	35.3490	42.4687
	esomeprazole	47.0544	55.9409	25.0216	41.6348	50.4232	34.8945
	esomeprazole	60.9746	55.0226	27.8176	41.3190	48.4688	36.9880
PPI	esomeprazole	62.2440	55.0524	25.1606	41.8928	55.6276	32.1850
	naproxen	32.6953	46.9447	32.6882	44.4375	51.8637	40.1226
	naproxen	31.4638	49.2903	31.3601	43.8162	52.1218	42.3574
	naproxen	29.4381	40.6523	34.4090	39.9886	41.2596	44.9445
	naproxen	32.8870	40.1969	33.9068	38.0396	39.9851	41.1872
	naproxen	39.7063	44.2710	33.0910	41.5705	52.5645	40.0741
	Ibuprofen	31.1749	44.6094	28.1189	34.8500	53.1838	40.7460
	Ibuprofen	28.0718	38.0893	26.1736	32.9494	52.2150	42.2240

	Ibuprofen	36.9724	40.8008	28.1771	32.7083	56.3140	43.4661
	Ibuprofen	34.5800	35.1149	27.9475	34.0148	54.0692	40.6065
	Ibuprofen	35.7916	40.6696	27.6902	33.0587	57.0970	40.2516
	diclofenac	35.5897	40.9747	29.7475	39.6789	54.3971	39.0340
	diclofenac	37.0628	40.3067	25.6118	37.0969	55.9325	37.3509
NASIDs	diclofenac	37.1851	43.4543	27.9226	36.3202	53.5008	35.1542
	diclofenac	37.2039	41.5786	27.7606	37.6986	55.9763	36.2794
	diclofenac	37.4930	40.7832	27.0342	36.6512	54.9517	36.9546
